# ALDH1A1 Inhibits Chicken Preadipocytes’ Proliferation and Differentiation via the PPARγ Pathway In Vitro and In Vivo

**DOI:** 10.3390/ijms21093150

**Published:** 2020-04-29

**Authors:** Jing Zhang, Bolin Cai, Manting Ma, Wei Luo, Zipeng Zhang, Xiquan Zhang, Qinghua Nie

**Affiliations:** 1 Department of Animal Genetics, Breeding and Reproduction, College of Animal Science, South China Agricultural University, Guangzhou 510642, Guangdong, China; jane_zhang233@hotmail.com (J.Z.); bolincai@stu.scau.edu.cn (B.C.); mamanting@stu.scau.edu.cn (M.M.); louwei198891@163.com (W.L.); zzp13226665505@126.com (Z.Z.); xqzhang@scau.edu.cn (X.Z.); 2 Guangdong Provincial Key Lab of Agro-Animal Genomics and Molecular Breeding, and Key Laboratory of Chicken Genetics, Breeding and Reproduction, Ministry of Agriculture, Guangzhou 510642, Guangdong, China; 3 National-Local Joint Engineering Research Center for Livestock Breeding, Guangzhou 510642, Guangdong, China

**Keywords:** ALDH1A1, preadipocytes, proliferation, differentiation, adipogenesis, PPARγ pathway, chicken

## Abstract

*ALDH1A1* (aldehyde dehydrogenase 1A1) is a crucial protein in retinoids’ metabolism, and the lack of *ALDH1A1* inhibits the fat deposition in mice. However, whether *ALDH1A1* has a similar effect on chickens’ fat-depot is still unknown. In this study, we investigate the role of *ALDH1A1* in chickens’ adipogenesis. The immortalized chicken preadipocyte 1 (ICP1) cell line and chicken primary preadipocytes isolated from abdominal fat were used to perform a series of experiments in vitro to elucidate the effects of *ALDH1A1*. In addition, lentivirus was used to verify the results of cell experiments in vivo. The data showed that overexpression of *ALDH1A1* significantly weakened the proliferation of preadipocytes and suppressed the differentiation of preadipocytes through the PPARγ pathway, and the knockdown experiments had the opposite results. Moreover, chickens injected with overexpression lentivirus had higher abdominal fat percentage, a bigger size of lipid droplets, and higher triglyceride content in abdominal fat, and chickens injected with interfering lentivirus had the opposite situation. We proved that *ALDH1A1* not only inhibited the proliferation and differentiation of chickens’ preadipocytes in vitro, but also inhibited the fat-depot of chickens in vivo, which was completely opposite the function of *ALDH1A1* in mice, indicating that *ALDH1A1* may have a different mechanism that is still unknown.

## 1. Introduction

Adipocyte differentiation is thought to be regulated by a series of transcription factors, including the C/EBPs protein family [1,2,3] and peroxisome proliferator-activated receptor g (PPARγ) [4]. On the one hand, retinoic acid (RA), the main product of aldehyde dehydrogenase 1A1 (ALDH1A1), inhibits adipogenesis through these transcription factors in mammals [5,6]. On the other hand, RA inhibits adipogenesis by binding to the retinoic acid receptor (RAR) [7,8,9]. Retinaldehyde dehydrogenases participate in the determination of cellular concentrations of free retinaldehyde by oxidizing retinaldehyde to RA [10].

*ALDH1A1* is a member of the ALDH gene family, which plays an important role in vitamin A (retinol) and carotenoid metabolism, catalyzing a series of reactions and maintaining the normal functions of animals [11]. According to the National Center of Biotechnology Information (NCBI) data, the coding sequences (CDS) of *ALDH1A1* in chicken contain 1529 bases, encoding 509 amino acids, with a total of 16 exons (www.ncbi.nlm.nih.gov/nuccore/NM_204577.4).

The aldehyde dehydrogenase 1 family catalyzes the reaction of vitamin A and its metabolites’ conversion, including retinaldehyde and retinoic acid [12]. ALDH1A1 is a key to the second step of the retinol metabolic pathway (vitamin A metabolism), which is the conversion of retinaldehyde to retinoic acid [13]. Retinol and its metabolites regulate other genes by activating some nuclear receptors, including the RAR and the retinoid X receptor (RXR), and releasing transcriptional factors, or binding to an RA response element and regulating the expression of target genes [14,15,16,17]. Thus, the ability on keep the balance between retinol and its metabolites can make ALDH1A1 involved in regulating other crucial genes that participate in important biological phenomenon like adipogenesis [18,19,20].

**Figure 1:**
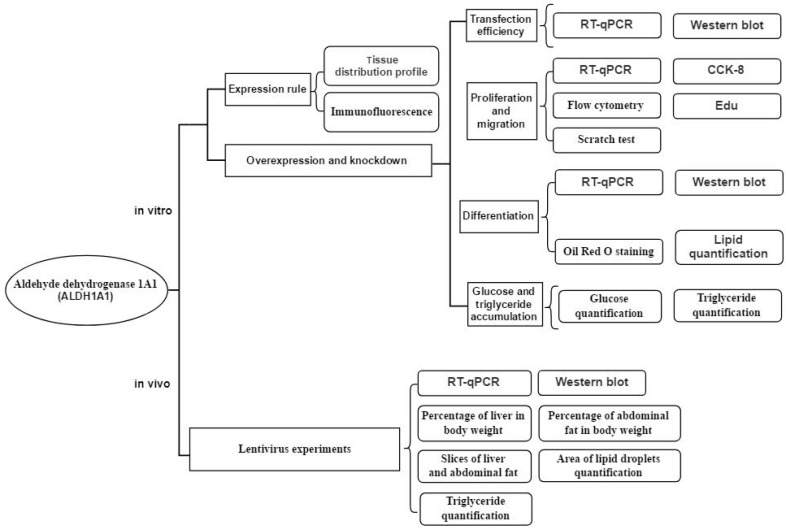
The workflow of the experiments on aldehyde dehydrogenase 1A1 (*ALDH1A1*). The experimental flow diagram for the *ALDH1A1* experiments in vitro and in vivo.

In recent years, *ALDH1A1* has been found to be involved in adipogenesis through vitamin A metabolism. Research has found that *ALDH1A1* deficiency inhibited high-fat diet-induced adipogenesis. At the same time, researchers observed that the lack of *ALDH1A1* prevented the visceral fat deposition induced by a high-fat diet in female mice, but in males, this effect was not as great as that of females, which meant *ALDH1A1* had different influences on male and female mice [18]. Other studies have shown that the lack of *ALDH1A1* induces a brown adipose tissue-like transcriptional program in white adipose tissue, promoting uncoupled respiration and adaptive thermogenesis, limiting weight gain in obese mice and improving glucose homeostasis [19]. Moreover, researchers found that *PPARγ* expression was significantly suppressed by zinc-finger protein 423 (*ZFP423*) reduction, and this change induced adipogenesis damage in *ALDH1A1*-deficient adipocytes. In their studies, they expressed *ALDH1s* in *ALDH1A1*^−/−^ cells and found a significant correlation between *ALDH1s* (including *ALDH1A1* and *ALDH1A3*), especially *ALDH1A1*, *ZFP423*, and *PPAR*γ expression levels. Otherwise, with low concentrations of RA, *PPAR*γ expression in *ALDH1A1*^−/−^ cells were partially recovered, indicating that *ALDH1A1*’s effects were dependent on RA production [20]. However, whether *ALDH1A1* plays a similar role in chicken is still unknown. To answer this question, we aimed to understand the function of *ALDH1A1* in chickens’ adipogenesis. In our study, we tried to find out whether *ALDH1A1* improved or inhibited the proliferation and differentiation of preadipocytes in vitro and whether it accelerated or impeded the fat-depot in chickens. The experimental flow is shown below (Figure 1).

## 2. Results

### 2.1. ALDH1A1 Expression in Chickens

**Figure 2:**
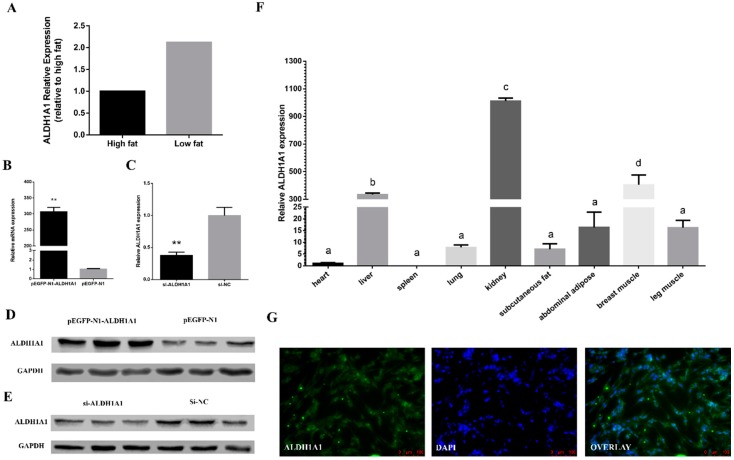
*ALDH1A1* expression in chickens. (**A**) The mRNA level of *ALDH1A1* in high abdominal fat chickens and low abdominal fat chickens. (**B**–**E**) The *ALDH1A1* mRNA (*n* = 6) and protein level (*n* = 3) in preadipocytes differentiated for five days after being transfected with the overexpression vector and small interfering RNA (siRNA), respectively. (**F**) The mRNA level of *ALDH1A1* in different tissues of seven weeks Xinghua chickens. Data were compared with the *ALDH1A1* mRNA expression of heart (*n* = 6). (**G**) *ALDH1A1* immunofluorescence of chicken primary preadipocytes in proliferation, scale bar: 100 μm. In all panels, the results are shown as the mean ± S.E.M., and the data are representative of three independent assays. The statistical significance of the differences between means was assessed using the unpaired Student’s *t*-test (* *p* < 0.05; ** *p* < 0.01) and Duncan’s test (*p* < 0.05).

The previous transcriptome sequencing experiments showed that the mRNA expression levels of *ALDH1A1* in high abdominal fat percentage chickens and low abdominal fat percentage chickens had significant differences [21]. *ALDH1A1* is highly expressed in low abdominal fat chickens’ abdominal fat, which indicated that *ALDH1A1* might play a negative role in the fat-depot in chickens (Figure 2A). To further investigate the function of *ALDH1A1*, the overexpression vector and small interfering RNA (siRNA) of *ALDH1A1* were produced. Their efficiency was detected by real-time quantitative polymerase chain reaction (RT-qPCR) and Western blot in chicken primary preadipocytes differentiated for five days (Figure 2B–E).

In addition, six chickens in seven weeks were slaughtered, and the tissues, including heart, liver, spleen, lung, kidney, abdominal fat, subcutaneous fat, breast muscle, and leg muscle, were collected and used to detect the mRNA level of *ALDH1A1* (Figure 2F). The data showed that *ALDH1A1* was expressed highest in kidney and decreased in the breast muscle, liver, abdominal fat, leg muscles, lungs, subcutaneous fat, heart, and spleen in turn. Moreover, after immunofluorescence staining, *ALDH1A1* was found to be expressed in both cell nucleus and cytoplasm (Figure 2G).

### 2.2. ALDH1A1 Represses Chicken Preadipocytes’ Proliferation and Migration

**Figure 3:**
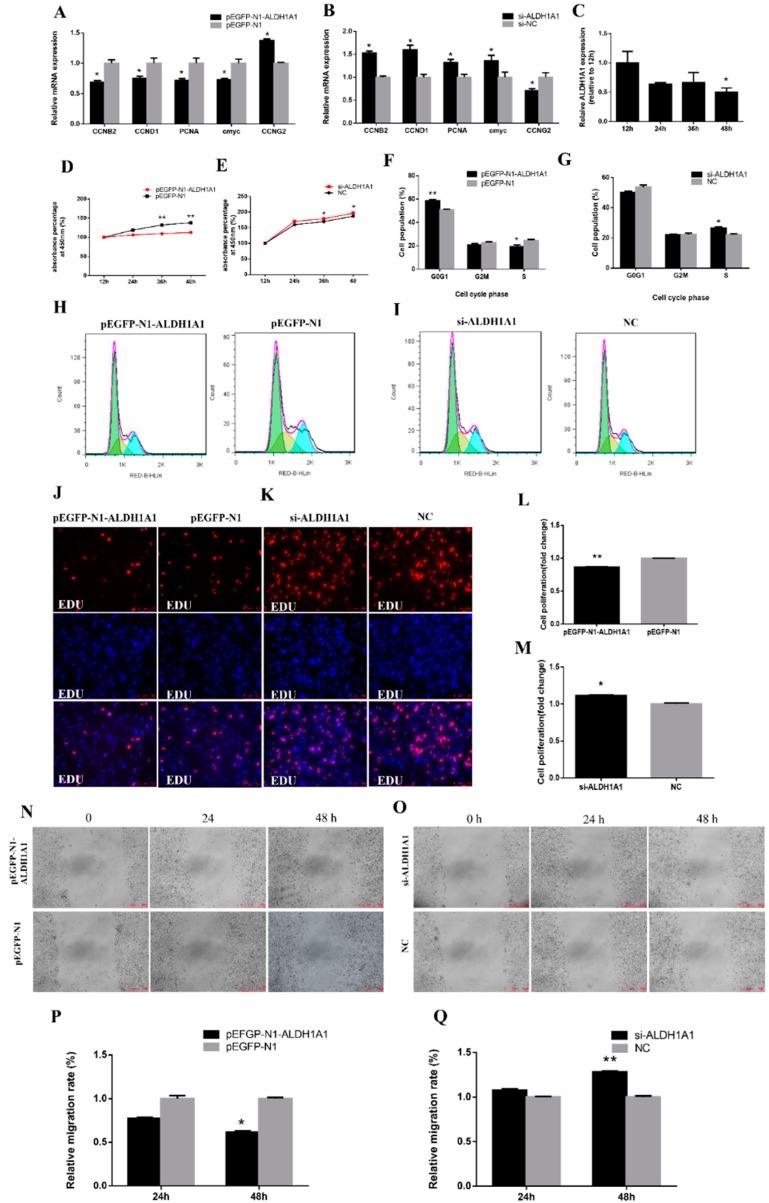
*ALDH1A1* represses chicken preadipocytes’ proliferation. (**A**,**B**) The mRNA expression of the cell cycle related gene after transfection for 48 h in chicken primary preadipocytes (*n* = 6). (**C**) The relative mRNA expression of *ALDH1A1* during proliferation in immortalized chicken preadipocyte 1 (ICP1) was measured by real-time quantitative polymerase chain reaction (RT-qPCR) using Dunnett’s test (*n* = 6, * *p* < 0.05; ** *p* < 0.01). (**D**,**E**) Cell growth relative to the “12 h” group was measured following the transfection of the overexpression vector of ALDH1A1 and siRNA of ALDH1A1 in ICP1 (*n* = 6). (**F**–**I**) Cell cycle analysis of ICP1 48 h after overexpression and inhibition of ALDH1A1, using propidium iodide staining for DNA content (*n* = 6). (**J**,**K**) Proliferation of transfected ICP1 was assessed by 5-ethynyl-2′-deoxyuridine (EdU) incorporation, scale bar: 100 μm. (**L**,**M**) Proliferation rates of ICP1 with *ALDH1A1* overexpression and inhibition (*n* = 6). (**N**,**O**) The scratch test detected the effect of *ALDH1A1* on cell migration, scale bar: 500 μm. (**P**,**Q**) Migration rate of ICP1 with *ALDH1A1* overexpression and inhibition (*n* = 6). In all panels, data are presented as the mean ± S.E.M. of three biological replicates. Statistical significance of differences between means was assessed using an unpaired Student’s *t*-test (* *p* < 0.05; ** *p* < 0.01) or Dunnett’s test (* *p* < 0.05; ** *p* < 0.01).

To explore the functions of *ALDH1A1*, we performed overexpression and knockdown experiments to assess its role in cell proliferation and viability. In immortalized chicken preadipocyte 1 (ICP1), we detected the mRNA expression of cell cycle-related gene, such as *CCNB2*, *CCND1*, *PCNA*, *cmyc*, and *CCNG2*. It was found that *CCNB2*, *CCND1*, *PCNA*, and *cmyc* were significantly downregulated when transfected with the overexpression vector of *ALDH1A1* and were significantly upregulated when transfected with si-*ALDH1A1.* Meanwhile, *CCNG2* was remarkably upregulated, while *ALDH1A1* was overexpressed and was reduced with si-*ALDH1A1* transfection (Figure 3A,B).

We detected the mRNA expression of *ALDH1A1* during proliferation in chicken primary preadipocytes, and the data showed that *ALDH1A1* expression showed a downward trend in 12 h, 24 h, 36 h, and 48 h of proliferation, suggesting that *ALDH1A1* may inhibit preadipocytes’ proliferation (Figure 3C). In addition, the Cell Counting Kit-8 (CCK-8) assay showed that cell proliferation was significantly repressed after 36 h and 48 h of transfection of the overexpression vector of *ALDH1A1* compared with the control group. Conversely, cell proliferation was significantly increased when transfected with siRNA of *ALDH1A1* (Figure 3D,E). Flow cytometry was used to detect the cell cycle status. It was found that overexpression of *ALDH1A1* significantly increased the number of cells staying in the G0/G1 phase and significantly reduced the number of cells remaining in the S phase, while interfering *ALDH1A1* expression remarkably increased the number of cells remaining in the S phase (Figure 3F–I). Moreover, the EdU (5-ethynyl-2′-deoxyuridine) assay also demonstrated that *ALDH1A1* overexpression significantly repressed chicken primary preadipocytes’ viability, while its inhibition promoted their proliferation (Figure 3J–M). In the scratch test, chicken primary preadipocytes transfected with the overexpression vector showed more activity in cell migration and showed the opposite situation while transfected with siRNA (Figure 3N–Q). All assays proved that *ALDH1A1* inhibited the proliferation and migration abilities of preadipocytes.

### 2.3. ALDH1A1 Represses Chicken Preadipocytes’ Differentiation through the PPARγ Pathway

**Figure 4:**
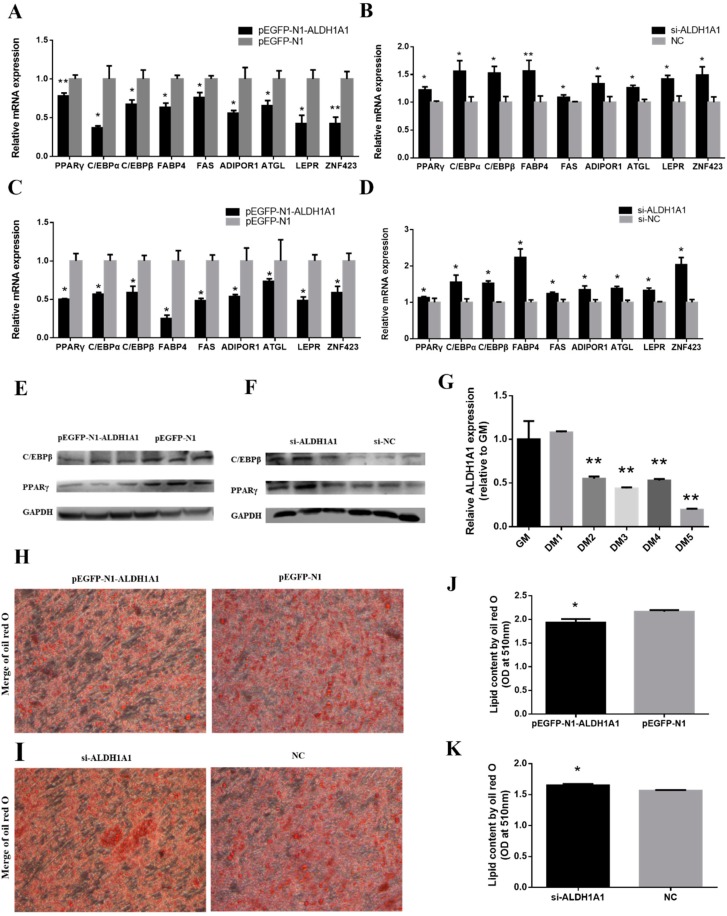
*ALDH1A1* represses chicken preadipocytes’ differentiation through the *PPARγ* pathway. (**A**,**B**) Relative mRNA levels of adipocyte differentiation-related genes after transfection in primary preadipocytes during differentiation, determined by RT-qPCR at 48 h post-transfection (*n* = 6). (**C**,**D**) Relative mRNA levels of adipocyte differentiation-related genes after transfection in ICP during differentiation, determined by RT-qPCR at 48 h post-transfection (*n* = 6). (**E**,**F**) The protein level of adipocyte differentiation-related genes after transfection and three days differentiation in primary preadipocytes (*n* = 3). (**G**) The *ALDH1A1* relative expression of primary preadipocytes during differentiation for five days was detected by RT-qPCR used Dunnett’s test (*n* = 6, * *p* < 0.05; ** *p* < 0.01). (**H**–**K**) Oil Red O test and lipid droplet quantification in primary preadipocytes after differentiation for five days (*n* = 6), scale bar: 50 μm. In all panels, data are presented as the mean ± S.E.M. of three biological replicates. The statistical significance of differences between means was assessed using an unpaired Student’s *t*-test (* *p* < 0.05; ** *p* < 0.01) or Dunnett’s test (* *p* < 0.05; ** *p* < 0.01).

To further investigate the potential roles of *ALDH1A1*, chicken primary preadipocytes and the ICP cell line were induced to differentiate for three days in vitro, and the mRNA level of some preadipocyte differentiation-related genes was measured by RT-qPCR, including *PPARγ*, *C/EBPα*, *C/EBPβ*, *FABP4*, *FAS*, *LEPR*, *ATGL*, *ZNF423*, and *ADIPOR1*. The protein levels of PPARγ and C/EBPβ in chicken primary preadipocytes differentiated for three days were measured by Western blot at the same time. The mRNA expressions of these relative genes were all significantly downregulated in the *ALDH1A1* overexpression vector transfected cells compared to control cells, especially the gene in the *PPARγ* pathway, including *PPARγ*, *C/EBPα*, *C/EBPβ*, *FABP4*, and *ZNF423* (Figure 4A,C). Conversely, inhibition of *ALDH1A1* promoted their expression (Figure 4B,D). The protein levels of PPARγ and C/EBPβ in chicken primary preadipocytes also showed similar results (Figure 4E,F). In addition, we detected the *ALDH1A1* mRNA expression during differentiation and found that the expression of *ALDH1A1* was downregulated from the second day, and it was significantly different from that of the proliferative stage (Figure 4G). Meanwhile, we used the Oil Red O test to analyze the lipid droplet content in different treatment groups. It was found that overexpression of *ALDH1A1* could observably depress the lipid droplet depot in adipocytes, and inhibition of *ALDH1A1* had the opposite effect (Figure 4H–K). All data above could prove that *ALDH1A1* was involved in the differentiation of chicken preadipocytes and had a suppressive effect on preadipocytes’ differentiation.

### 2.4. ALDH1A1 Inhibits Glucose and Triglyceride Accumulation in Chicken Preadipocytes

**Figure 5:**
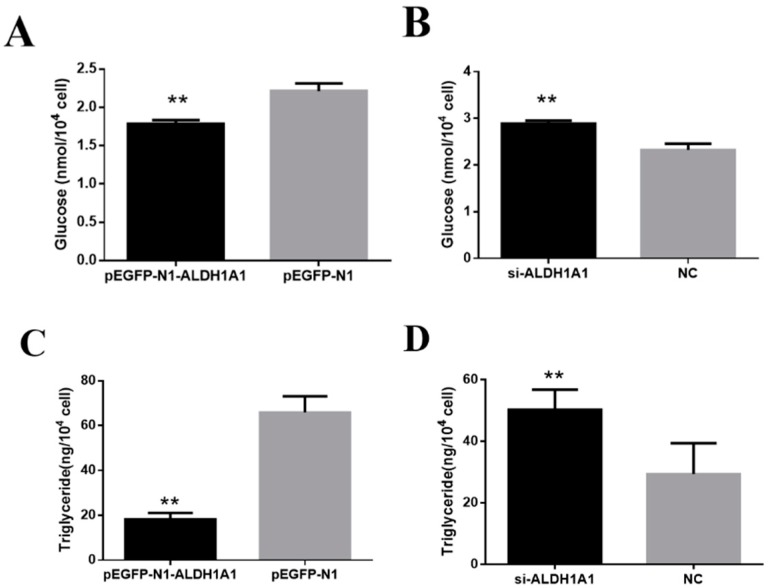
*ALDH1A1* inhibits glucose accumulation in chicken preadipocytes. (**A**,**B**) Glucose content in chicken primary preadipocytes after transfection and differentiation for three days (*n* = 6). (**C**,**D**) Triglyceride accumulation in the preadipocytes after differentiation for five days was detected under 420 nm (*n* = 6). Statistical significance of the differences between the means was assessed using an unpaired Student’s *t*-test (** *p* < 0.01) vs. negative control (NC).

After transfection of the primary preadipocytes with the overexpression vector and the siRNA, the cells were induced to differentiate for three days, and the glucose content was measured. The cells were collected by protease, and ultrasonic wave was used to destroy the biological membrane under the low-temperature environment, in order to release the glucose inside the cytoplasm and accurately detected the glucose content in cells. It was found that overexpression of *ALDH1A1* could significantly inhibit the accumulation of glucose in the cells, and the interference of *ALDH1A1* expression showed the opposite result, accelerating the glucose deposition in cells (Figure 5A,B). These results demonstrated that, generally, *ALDH1A1* had a certain inhibitory effect on glucose accumulation in differentiated preadipocytes and, to a certain extent, confirmed that *ALDH1A1* had a negative effect on fat deposition in chickens. 

Moreover, we analyzed the triglyceride content after five days of differentiation of adipocytes, and the data showed that *ALDH1A1* repressed the triglyceride accumulation in the preadipocytes during early differentiation (Figure 5C,D), demonstrating that *ALDH1A1* could inhibit the accumulation of triglyceride in preadipocytes.

### 2.5. ALDH1A1 Inhibits Fat-Depot in Chickens

To find out whether *ALDH1A1* inhibited fat-depot in chickens, we injected lentivirus into 42 two-weeks-old chickens at liver and peritoneal cavity. Chickens were divided into four groups, including the pWPXL-N1-*ALDH1A1* group (*n* = 9), the pWPXL group (*n* = 9), the si-*ALDH1A1* group (*n* = 12), and the si-NC group (*n* = 12) and were slaughtered at three weeks old. The data of the body weight, the mass of liver, and the mass of abdominal fat are shown in Table S1. Liver and abdominal fat were collected and were used to detect the size of the lipid droplet, triglyceride content, and the mRNA and protein level of ALDH1A1. All chickens were female and were under free feed intake.

**Figure 6:**
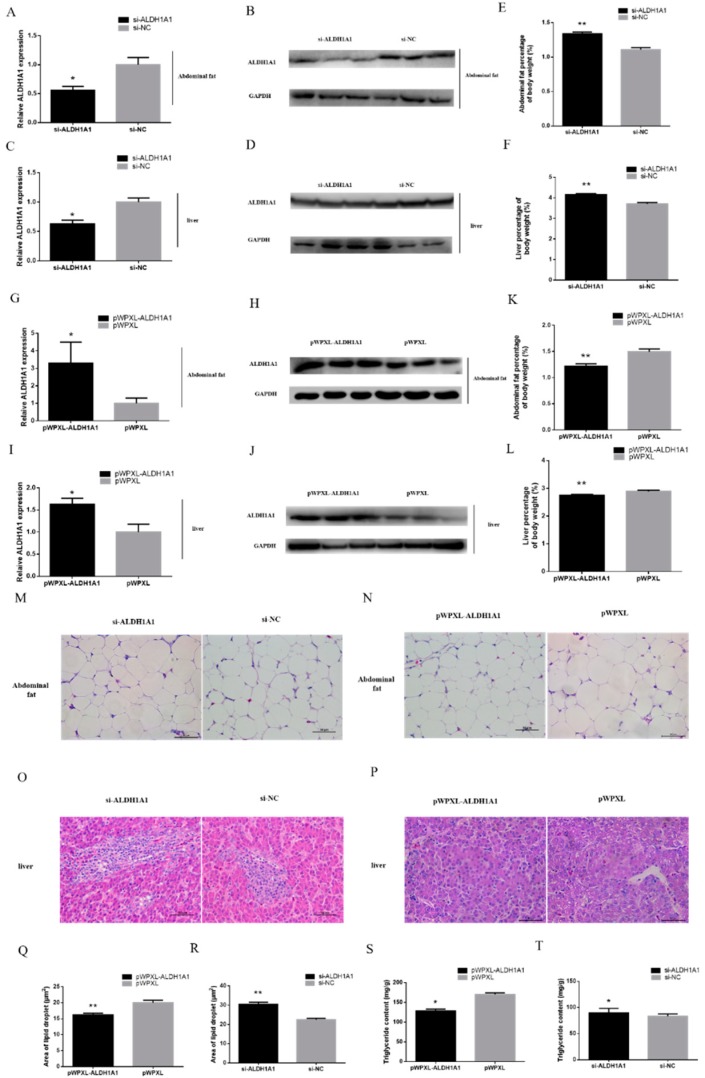
*ALDH1A1* inhibits fat-depot in chickens. (**A**–**D**) *ALDH1A1* mRNA relative expression (*n* = 12) and protein level in abdominal fat and liver after interfering lentivirus injection for a week. (**E**,**F**) Abdominal fat percentage and mass of liver after interfering lentivirus injection for a week (*n* = 12). (**G**–**J**) *ALDH1A1* mRNA relative expression (*n* = 9) and protein level in abdominal fat and liver after overexpression lentivirus injection for a week. (**K**,**L**) Abdominal fat percentage and mass of liver after overexpression lentivirus injection for a week (*n* = 9). (**M**–**P**) The slices of abdominal fat and liver from different groups, scale bar: 50 μm. (**Q**,**R**) The area of lipid droplets in abdominal fat from different groups analyzed by ImageJ. (**S**,**T**) The triglyceride accumulation in abdominal fat from different groups. In all panels, data are presented as the mean ± S.E.M. of three biological replicates. The statistical significance of differences between means was assessed using an unpaired Student’s *t*-test (* *p* < 0.05; ** *p* < 0.01) vs. NC.

The result of Western blot and RT-qPCR proved that the administration of lentivirus did reduce or increase the expression of *ALDH1A1* in liver and abdominal fat (Figure 6A–D,G–J).

We found that *ALDH1A1* could inhibit the fat-depot in abdominal fat. The chickens injected with interference lentivirus had a higher abdominal fat percentage and a bigger liver, while overexpression of *ALDH1A1* showed the opposite situation (Figure 6E,F,K,L). Moreover, we captured the slices of their abdominal fat and calculated the area of lipid droplets by ImageJ, and the results showed that chickens injected with interference lentivirus had bigger lipid droplets and a small tendency for adiposis hepatica. However, the chickens injected with overexpression lentivirus had smaller lipid droplets, indicating that *ALDH1A1* expression could significantly reduce the fat deposition in chickens (Figure 6M–R). In addition, we analyzed the triglyceride content in abdominal fat and found that *ALDH1A1* suppressed the triglyceride accumulation (Figure 6S,T).

## 3. Discussion

Chicken is the second most consumed meat in China, and the quality of chicken has become a more important tissue that determines the commercial value of chicken. Abdominal fat, one of the important issues that affects the price of chicken, is significantly influenced by RA [22]. Reports showed that RA could significantly inhibit the adipogenesis in mice through the PPARγ pathway [23]. *ALDH1A1* is the crucial protease in the reaction of the conversion of retinol to RA [16], indicating that *ALDH1A1* may play an important role in fat deposition. The previous study revealed the role of *ALDH1A1* in preadipocytes’ proliferation and differentiation in mice. Moreover, in recent years, many reports also proved that *ALDH1A1* promoted the fat-depot in mice [20,24]. The nuclear hormone receptor *PPAR*γ promotes adipogenesis [25], and it was found that *PPAR*γ was affected by *ZFP423* expression, showing prominent reduction in *ALDH1A1* knock-down adipocytes [20].

Our findings were partly based on the function of *ALDH1A1* on fat-depot, but surprisingly found that *ALDH1A1* had a completely different influence on chicken fat-depot. In our previous RNA sequencing data, we found that *ALDH1A1* had higher expression in chickens with lower abdominal fat percentage. In subsequent experiments, we detected the mRNA level of *ALDH1A1* in primary preadipocytes during proliferation and differentiation. It was found that *ALDH1A1* was downregulated during the proliferation and early differentiation stage. To further investigate the biological functions of *ALDH1A1*, we performed a series of in vitro and in vivo experiments and found that *ALDH1A1* inhibited the proliferation and differentiation of preadipocytes and the fat deposition in chicken.

To perform the in vitro experiments, we constructed an overexpression vector and synthesized siRNA to overexpress and knockdown the expression of *ALDH1A1*. With the series of experiments, our results showed that *ALDH1A1* was highly expressed in abdominal fat and expressed in both cell nucleus and cytoplasm. Moreover, in preadipocytes, *ALDH1A1* inhibited the genes promoting cell proliferation and upregulated genes suppressing cell proliferation [26,27]. The differentiation-related genes [28], including *PPAR*γ, C/*EBP*α, C/*EBP*β, *FABP4*, *FAS*, *LEPR*, *ATGL*, *ZNF423*, and *ADIPOR1*, were repressed as well. The process of adipogenesis is controlled by diverse factors, and one of the famous ones is *PPAR*γ, which is known to promote fat cell differentiation in vitro [29]. Upon activation, *PPAR*γ heterodimerizes with the retinoid X receptor, recruiting factors, combining with the peculiar DNA structure, so as to activate the adipogenesis target genes [30]. Interestingly, in a previous study, *ALDH1A1*′s deficiency significantly reduced the mRNA expression of *PPAR*γ and *ZNF423* in mice [20], which was different from the results of experiments on chicken. The differences indicated that one of the reasons that *ALDH1A1* had a different effect between mice and chicken might be the changes of *PPAR*γ and *ZNF423*. 

In our study, on the one hand, overexpression of *ALDH1A1* significantly reduced the number of cells in the S phase, while transfection with siRNA remarkably increased the number of cells in the S phase, proving that *ALDH1A1* inhibited the cell cycle. On the other hand, the EdU and CCK-8 assays showed that *ALDH1A1* also downregulated the proliferative activity of preadipocytes. In addition, we found that *ALDH1A1* inhibited the accumulation of lipogenesis. The cell transfected with pEGFP-N1-*ALDH1A1* had a small size of the lipid droplets and a lower content of triglyceride, while the cell transfected with siRNA had the completely opposite situation, which meant that *ALDH1A1* blocked the lipogenesis in vitro. Above all, we drew the conclusion that *ALDH1A1* inhibited the proliferation and differentiation of preadipocytes, which blocked the adipogenesis in chicken.

To further investigate whether *ALDH1A1* inhibited the adipogenesis in vivo, we performed the lentivirus assay, injecting lentivirus into chickens’ liver and enterocoelia [31,32,33] and identified the transfection efficiency by RT-qPCR and Western blot. We used the tissues, which were successfully injected by lentivirus, to perform the following experiments. Moreover, we detected the abdominal fat percentage, size of lipid droplets, mass of liver, and triglyceride content of abdominal fat. The results showed that *ALDH1A1* inhibited the adipogenesis in chickens.

## 4. Materials and Methods

### 4.1. Ethics Statement

Animal experiments in the research were under the rigorous supervision of the Administration of Laboratory Animals in Guangdong Province. All operations involved in animals aimed to reduce the pain of the animals. The experimental operations were approved by the Institutional Animal Care and Use Committee at South China Agricultural University (approval ID: SCAU#0011, 3 June 2019).

### 4.2. Experimental Animals and Tissues

In the laboratory, prior to transcriptome sequencing, one-hundred-fifty-eight 100 days old female chickens were supplied by Wens Co., Ltd. (Yunfu, China) with free water and food. Chickens were divided into two groups according to the abdominal fat percentage (high-fat group and low-fat group). The chickens were slaughtered at 100 days old after 12 h of fasting, and the abdominal fat was collected and stored in liquid nitrogen to avoid the degradation of RNA. The HiPure Total RNA Plus Mini Kit (Megen, Guangzhou, China) was used to exact the total RNA, and the RNA was sent to the Beijing Genomics Institution (Beijing, China) as the pooled samples for quality testing and RNA sequencing [21]. In this research, we chose *ALDH1A1*′s different expression data in abdominal fat. The data of these two groups are recorded in Table 1.

Six 7-weeks-old chickens were fed at South China Agriculture University (Guangzhou, China). Nine tissues, heart, liver, spleen, lung, kidney, abdominal fat, subcutaneous fat, breast muscle. and leg muscle, were collected and stored in liquid nitrogen immediately in order to protect the RNA from degrading. TRIzol reagent (TaKaRa, Kyoto, Japan) was used to extract the total RNA, and the PrimeScript RT Reagent Kit (TaKaRa, Kyoto, Japan) was used to synthesize cDNA to detect the mRNA level of *ALDH1A1*. The Bio-Rad Real-Time Detection Machine (Bio-Rad, Hercules, CA, USA) was utilized to process the RT-qPCR and calculate the fold change.

Forty-two chickens at one day old, which were bought from Yuhe Agriculture and Animal Husbandry Co., Ltd. (Zhuhai, China), were housed in the same room with free food and water. Overexpression lentivirus, control lentivirus, interference lentivirus, or interference negative control lentivirus (3 × 10^7^ infectious lentivirus particles per chicken) were delivered by liver injection and intraperitoneal injection to 2-weeks-old chickens. The chickens were slaughtered at 3 weeks old, and the liver and abdominal fat were collected and used to detect the mRNA expression of *ALDH1A1*. Only tissues that had a significant difference from the negative control groups on ALDH1A1 mRNA expression were used for subsequent experiments. The selected tissues were used to analyze the abdominal fat percentage, mass of liver, triglyceride content, and protein level of *ALDH1A1*.

### 4.3. Lentivirus Production and Transduction

To generate lentivirus, the full-length sequence of *ALDH1A1* was inserted into pWPXL (Addgene, Watertown, MA, USA), and the recombinant lentiviral expression vectors were co-transfected with PMD 2.4G (Addgene, Watertown, MA, USA) and psPAX2 (Addgene, Watertown, MA, USA) into 293T cell by using Lipofectamin 3000 reagent (Gibco, Grand Island, NY, USA). The supernatant was collected after 48 h and 72 h of transfection, filtered through 0.45 μm PVDF membranes (Millipore, Boston, MA, USA), and concentrated by ultracentrifugation. The viral titer was evaluated by gradient dilution. The sequence of si-*ALDH1A1* was given to Hanbio Biotechnology (Shanghai, China) to produce interfering lentivirus. The viral titers of these lentiviruses were 5.0 × 10^8^ TU/mL and 5.2 × 10^8^ TU/mL, respectively.

### 4.4. Cell Culture

ICP1 cell culture: The ICP1 cell line was bought from Northeast Agricultural University (Harbin, China) and cultured with Dulbecco’s Modified Eagle Medium (DMEM, Gibco, Grand Island, NY, USA). Ten percent fetal bovine serum (FBS, Hyclone, Logan, UT, USA) and 0.2% penicillin and streptomycin (Invitrogen, Carlsbad, CA, USA) were supplemented in DMEM.

Chicken primary preadipocytes’ isolation and culture: Primary preadipocytes were isolated from the abdominal fat of 3 weeks old chickens. Firstly, the abdominal fat was dissected away from the enterocoelia and washed by using DMEM. Secondly, the abdominal fat was cut into pieces by surgery scissors and digested by collagenase I for 30 min at 37 °C in order to acquire a single-cell suspension. Then, the suspension was centrifuged at 1300× *g* to collect the preadipocytes. After this protocol, cells were seeded in petri dishes with complete medium. The ingredients in the complete medium of primary preadipocytes were the same as those for ICP1. 

The differentiation of preadipocytes was induced by DMEM/F12 medium supplemented with 0.2% penicillin/streptomycin and 0.05% sodium oleate [34,35,36,37].

All cells were cultured at 37 °C in a 5% CO_2_ humidified atmosphere.

### 4.5. RNA Isolation, Complementary DNA Synthesis, and RT-qPCR Analysis

RNA in tissues and cells was extracted by TRIzol reagent (TaKaRa, Kyoto, Japan) under the guidance of the manufacturer’s instructions. The quality of RNA was examined by 1.5% agarose gel electrophoresis and detected by Nanodrop 2000c spectrophotometer (Thermo, Waltham, MA, USA). The PrimeScript RT Reagent Kit (TaKaRa, Kyoto, Japan) was used to synthesize cDNA and get rid of genomic DNA (gDNA). The Bio-Rad Real-Time Detection Machine (Bio-Rad, Hercules, CA, USA) was utilized to detect and amplify cDNA with the iTaq Universal SYBR Green Supermix Kit (Toyobo, Osaka, Japan). *GAPDH* was used as the internal control. Data analysis was carried out with the comparative 2^−ΔΔCT^ method [38].

### 4.6. Primers

Premier Primer 5.0 (Premier Biosoft International, Palo Alto, CA, USA) and Oligo 7 (Molecular Biology Insights Inc., Cascade, CO, USA) were utilized to devise the sequences of primers. Primers were sent to Sangon Biotech (Shanghai, China) and Tsingke Biotech (Beijing, China) for synthesis. Primers used for vector construction are listed in Table S2. Primers for RT-qPCR are shown in Tables S3 and S4.

### 4.7. RNA Oligonucleotides, Plasmids Construction, and Lentivirus Construction

siRNA used for the knockdown of *ALDH1A1* was devised and sent to RiboBio (Guangzhou, China) for synthesis. The sequence of siRNA is listed in Table S5. For RNA oligonucleotides, a concentration of 100 nM was used.

For *ALDH1A1* overexpression plasmid construction, the full-length sequence of *ALDH1A1* was amplified from chicken abdominal fat cDNA by PCR and cloned into the expression plasmid, the pEGFP-N1 vector (Promega, Madison, WI, USA), by using the *NheI* and *XhoI* restriction sites.

For lentivirus, the same cDNA cloned before was cloned into the pWPXL vector (Promega, Madison, WI, USA) by using the *SmaI* and *MIuI* restriction sites. Then, the overexpression plasmid and the sequence of si-*ALDH1A1* were given to Hanbio Biotechnology (Shanghai, China) to produce overexpression and interfering lentivirus.

### 4.8. Western Blotting Assay

Radio immune precipitation assay buffer (Beyotime, Shanghai, China) supplemented with 10% phenylmethane sulfonyl fluoride protease inhibitor (Beyotime, Shanghai, China) was used to extract protein in tissues and cells under the guidance of the manufacturer’s direction. Proteins after denaturation were loaded into the sample holes of 10% SDS-PAGE and separated under a stable voltage. Nitrocellulose membranes (Whatman, Maidstone, UK) were put on the surface of 10% SDS-PAGE and transferred protein under a constant electricity. Then, antibodies were utilized to combine the antigens.

The antibodies and their dilutions used for Western blots were as follows: Rabbit Anti-ALDH1A1 antibody (GTX123973; GeneTex, San Antonio, TX, USA; 1:5000), Rabbit Anti-CEBP Beta Polyclonal Antibody (bs-1396R; Bioss, Beijing, China; 1:500) [39,40], Rabbit Anti-PPAR Gamma Polyclonal Antibody (bs-0530R; Bioss, China; 1:500), Rabbit Anti-Adiponectin Polyclonal Antibody (bs-0471R; Bioss, Beijing, China; 1:500) [41], Rabbit Anti-GAPDH (AB-P-R 001; Hangzhou Goodhere Biotechnology Co. Ltd., Hangzhou, China; 1:10,000), Goat Anti-rabbit IgG-HRP(BA1054; Boster, Wuhan, China; 1:10,000), and Peroxidase-goat Anti-mouse IgG (BA1051; Boster, Wuhan, China; 1:10,000).

### 4.9. EdU Assay

Cells seeded in 12 well plates were cultured to 70% density and then transfected. Forty-eight hours after transfection with siRNAs or the overexpression vector, the cells were fixed and stained with a C10310 EdU Apollo In Vitro Imaging Kit (RiboBio, China). A fluorescence microscope (TE2000-U; Nikon, Tokyo, Japan) was used to capture three randomly selected fields, and the ImageJ software (National Institutes of Health, Bethesda, MD, USA) was used to count the number of EdU-stained cells.

### 4.10. Analysis of the Cell Cycle

Flow cytometry (FCM) was used to detect the cell cycle of preadipocytes. Cells after different treatments were collected and stored with 70% ethanol for more than 2 h under −20 °C. Then, the preadipocytes were dyed with 50 μg/mL propidium iodide (Sigma, Saint Louis, MO, USA), 0.2% Triton X-100 (Sigma, Saint Louis, MO, USA), and 10 μg/mL RNase A (Takara, Kyoto, Japan). After that, a 37 °C incubator was used to incubate the stained cells for 30 min. A flow cytometer (BD Biosciences, Franklin Lakes, NJ, USA) was used to perform FCM, and the data were analyzed by FlowJo7.6 software (BD Biosciences, Franklin Lakes, NJ, USA).

### 4.11. CCK-8 Experiments

Preadipocytes were transferred to the 96 well culture plate and transfected with the overexpression vector and siRNA. A Model 680 Microplate Reader (Bio-Rad) was used to detect the absorbance of cells at 450 nm after being incubated with TransDetect CCK (TransGen Biotech, Beijing, China) for 1 h. The data were detected at 12 h, 24 h, 36 h, and 48 h after transfection [42]. 

### 4.12. Immunofluorescence Assay

Preadipocytes were seeded in 12 well culture plates and transfected with the overexpression vector and siRNA. After 48 h, preadipocytes were incubated with 4% formaldehyde for 20 min for fixation. Then, cells were washed by phosphate buffer saline (PBS) several times and the biological membrane damaged with 0.1% Triton X-100 for 15 min. After that, the preadipocytes were blocked by goat serum for 30 min and incubated with Rabbit Anti-ALDH1A1 antibody overnight at 4 °C. The Fluorescein (FITC)-conjugated AffiniPure Goat Anti-Rabbit IgG (H + L) (BS10950; Bioworld, USA; 1:50) was used to combine the antibody/antigen complex at normal temperature for 1 h. The preadipocytes’ nuclei were dyed by 4’,6-diamidino-2-phenylindole (DAPI) for 5 min, and a fluorescence microscope (Nikon, Tokyo, Japan) was utilized to capture the pictures of immunofluorescence. 

### 4.13. Oil Red O Staining and Quantification

Preadipocytes were seeded in 12 well culture plates. After transfection and differentiation for 5 days, differentiated preadipocytes were washed by PBS and incubated with 4% formaldehyde for 20 min for fixation. Then, differentiated preadipocytes were dyed with Oil Red O working solution (Solarbio, Beijing, China) under the direction of the manufacturer’s specification for 30 min at room temperature and washed five times with distilled water. Then, DAPI was added and incubated for 5 min. After washing, a fluorescence inverted light microscope (Leica DMi8, Wetzlar, Germany) was used to capture the images. In the end, the stain in the cells was extracted by isopropanol and analyzed at 510 nm absorbance with a NanoDrop 2000C Spectrophotometers (Thermo).

### 4.14. Triglyceride Quantification

Cells were seeded in 24 well plates and transfected when cells were cultured to 70% density. After 5 days of differentiation, cells were collected by 0.25% trypsin-EDTA (Gibco, USA), disrupted by an Ultrasonic Cell Crusher (Biolon, Shanghai, China), and the triglyceride content detected by using the Triglyceride Assay Kit (Solarbio, China), according to the manufacturer’s protocol.

### 4.15. Glucose Quantification

Cells were seeded in 12 well plates and transfected when cells were cultured to 70% density. After 3 days of differentiation, cells were collected and disrupted by ultrasonic waves at 4 °C. The cell suspension was collected and used to continue subsequent detection by the Glucose Assay Kit (Solarbio, China), according to the manufacturer’s protocol.

## 5. Conclusions

In summary, *ALDH1A1* not only inhibited the proliferation of preadipocytes, but also inhibited the differentiation of preadipocytes, blocking the adipogenesis in vitro. In addition, *ALDH1A1* had a similar effect on adipogenesis in vivo as well, which was completely different from the effect of *ALDH1A1* on mice, indicating that the molecular mechanism of *ALDH1A1* in adipogenesis may have a tremendous difference from that of mice, and it is worthwhile for us to make a further investigation.

## Figures and Tables

**Table 1:** Slaughter data of female chickens.

Groups	Body Weight (g)	Mass of Abdominal Fat (g)	Abdominal Fat Percentage (%)
High Fat	1659.93 ± 60.61	146.95 ± 8.58 **	8.83 ± 0.28 **
Low Fat	1472.63 ± 68.49	53.67 ± 4.35	3.62 ± 0.17

** Represents that the mass of abdominal fat and abdominal fat percentage of the high-fat group were significantly higher than low-fat group. The statistical significance of differences between means was assessed using an unpaired Student’s *t*-test (** *p* < 0.01).

## Supplementary Materials

Supplementary materials can be found at https://www.mdpi.com/1422-0067/21/9/3150/s1. Table S1: Data of slaughtered chickens used in the lentivirus experiments. Table S2: Primer used for vector construction. Table S3: Cell cycle gene primer and ALDH1A1 primer used for RT-qPCR. Table S4: Differentiation regulated gene primer used for RT-qPCR. Table S5: Oligonucleotides sequences in this study.
