# Supplementary material for: ALDH1A1 Inhibits Chicken Preadipocytes’ Proliferation and Differentiation via the PPARγ Pathway In Vitro and In Vivo
Source: Int J Mol Sci. 2020 Apr 29;21(9):3150. doi: 10.3390/ijms21093150 (PMC7246604; doi:10.3390/ijms21093150)
Supplement: Supplementary file 1 [file ijms-21-03150-s001.zip › Supplementary File/Table S1-5.docx]

**Table S1** Data of slaughter chickens used in lentivirus experiments

| Groups | number | Body weight (g) | Mass of abdominal fat (g) | Mass of liver (g) |
| --- | --- | --- | --- | --- |
| PWPXL-ALDH1A1 | 1 | 180.4 | 5 | 2.3 |
|  | 2 | 190.5 | 5.4 | 2.5 |
|  | 3 | 185.2 | 5.3 | 2.4 |
|  | 4 | 201.4 | 5.4 | 2.8 |
|  | 5 | 210.5 | 5.9 | 2.8 |
|  | 6 | 205.6 | 5.3 | 2.5 |
|  | 7 | 202.4 | 5.6 | 2.1 |
|  | 8 | 211.1 | 5.8 | 2 |
|  | 9 | 218.4 | 6 | 2.5 |
| PWPXL | 10 | 220.4 | 6 | 3.2 |
|  | 11 | 210 | 5.8 | 2.9 |
|  | 12 | 209.1 | 6.2 | 3.2 |
|  | 13 | 215.2 | 6 | 3.5 |
|  | 14 | 225.3 | 6.8 | 3.7 |
|  | 15 | 201.2 | 5.9 | 3.2 |
|  | 16 | 190.9 | 5.6 | 3.2 |
|  | 17 | 205.8 | 5.7 | 2.4 |
|  | 18 | 194.5 | 6.1 | 2.7 |
| si-ALDH1A1 | 19 | 240.2 | 10.4 | 3.2 |
|  | 20 | 266.6 | 11 | 3.4 |
|  | 21 | 238.7 | 9.6 | 3.5 |
|  | 22 | 245 | 10.7 | 3 |
|  | 23 | 274.5 | 11.1 | 3.4 |
|  | 24 | 278.3 | 10.8 | 3.6 |
|  | 25 | 258.1 | 11 | 3.6 |
|  | 26 | 250.1 | 10.5 | 3.7 |
|  | 27 | 264 .2 | 10.8 | 3.8 |
|  | 28 | 258.8 | 10.9 | 3.2 |
|  | 29 | 270.3 | 11.2 | 3.5 |
|  | 30 | 254.5 | 10.9 | 3.5 |
| NC | 31 | 258.2 | 10.1 | 2.9 |
|  | 32 | 250.2 | 8.6 | 3.1 |
|  | 33 | 276.4 | 11 | 3.1 |
|  | 34 | 278.4 | 10.4 | 3.2 |
|  | 35 | 245.6 | 9.2 | 2.7 |
|  | 36 | 266.3 | 9.8 | 2.5 |
|  | 37 | 233.5 | 8.8 | 2.3 |
|  | 38 | 254 .8 | 8.6 | 3.3 |
|  | 39 | 277.1 | 11.5 | 3.2 |
|  | 40 | 240 | 9 | 2.6 |
|  | 41 | 263.3 | 9.2 | 2.9 |
|  | 42 | 271.2 | 9.2 | 2.7 |

**Table S2.** *ALDH1A1* primer and cell cycle genes’ primers used for RT-qPCR.

| **Gene name** | **Primer sequences (5’-3’)** | **Size**  **(bp)** | **Annealing temperature (℃)** | **Accession number** |
| --- | --- | --- | --- | --- |
| *ALDH1A1* | F: GAAGTTCAATTAAAATCTGGGG | 151 | 56 | NM_204577.4 |
|  | R: ATCTTTGAGTGGTTCTGGCA |  |  |  |
| *GAPDH* | F: CAACTTTGGCATTGTGGAGG | 130 | 56 | NM_204305.1 |
|  | R: CGCTGGGATGATGTTCTGG |  |  |  |
| *CCNB2* | F: CAGTAAAGGCTACGAAAG | 133 | 58 | NM_001004369.1 |
|  | R: ACATCCATAGGGACAGG |  |  |  |
| *CCND1* | F: CAGAAGTGCGAAGAGGAAGT | 188 | 58 | NM_205381.1 |
|  | R: CTGATGGAGTTGTCGGTGTA |  |  |  |
| *PCNA* | F: GTGCTGGGACCTGGGTT | 217 | 58 | NM_204170.2 |
|  | R: CGTATCCGCATTGTCTTCT |  |  |  |
| *cmyc* | F: TACCTGCACGACCTGGGA | 158 | 58 | NM_001030952.1 |
|  | R: TCGGTTGTTGCTGATCTGTTT |  |  |  |
| *CCNG2* | F: TGCCAACAATACCAGAGG | 260 | 58 | XM_420475.5 |
|  | R: TACAGAATACCACAATCCC |  |  |  |

**Table S3.** Primers used for vector construction.

| **Primer name** | **Primer sequences (5’-3’)** | **Size(bp)** | **Annealing temperature (℃)** |
| --- | --- | --- | --- |
| pEGFP-N1-*ALDH1A1* | F: CTA**GCTAGC**ATGAAGAAGCAAGGCTCACC | 1530 | 56 |
|  | R: CCG**CTCGAG**TTATGAGTTCTTCTGTGGGATT |  |  |
| pWPXL-  *ALDH1A1* | F: CGA**CGCGT**ATGAAGAAGCAAGGCTCACC | 1530 | 56 |
|  | R: TCC**CCCGGG**TTATGAGTTCTTCTGTGGGATT |  |  |

Sequences in bold represent the enzyme cutting sites.

**Table S4.** Differentiation regulated genes’ primers used for RT-qPCR.

| **Gene name** | **Primer sequences (5’-3’)** | **Size**  **(bp)** | **Annealing temperature (℃)** | **Accession number** |
| --- | --- | --- | --- | --- |
| *GAPDH* | F: CAACTTTGGCATTGTGGAGG | 130 | 56 | NM_204305.1 |
|  | R: CGCTGGGATGATGTTCTGG |  |  |  |
| *PPAR*γ | F: TCCTTCCCGCTGACCAAA | 227 | 60 | NM_001001460.1 |
|  | R: TCCTGCACTGCCTCCACA |  |  |  |
| C/*EBP*α | F: GACAAGAACAGCAACGAGTACCGC | 195 | 56 | NM_001031459.1 |
|  | R: CCTGAAGATGCCCCGCAGAGT |  |  |  |
| C/*EBP*β | F: GCGGACTGTTTGGCTGCTCT | 220 | 60 | NM_205253.2 |
|  | R: CGGGTGAGGCTGATGTAGGTGT |  |  |  |
| *ADIPOR1* | F: GACAAGAACAGCAACGAGTACCGC | 110 | 60 | NM_001031027.1 |
|  | R: CCTGAAGATGCCCCGCAGAGT |  |  |  |
| *ADIPOQ* | F: GACAAGAACAGCAACGAGTACCGC | 189 | 64 | NM_206991.1 |
|  | R: CCTGAAGATGCCCCGCAGAGT |  |  |  |
| *FABP4* | F: ATGTGCGACCAGTTTGTG | 222 | 56 | NM_204290.1 |
|  | R: TTTGCCATCCCACTTCTG |  |  |  |
| *FAS* | F: CGCAGGCATAGCAGGAAA | 180 | 60 | NM_205155.2 |
|  | R: CCAAAGAAGGAGGCATCAA |  |  |  |
| *LEPR* | F: CCAACCCTTCCTTGCTAA | 182 | 60 | NM_204323.1 |
|  | R: GCCTTCAACCCAACATTC |  |  |  |
| *LPL* | F: CCAAGGTAGACCAGCCATTC | 154 | 60 | NM_205282.1 |
|  | R: TGCTCCAGGCACTTCACA |  |  |  |
| *ATGL* | F: TGTCCAAAGAAGCACGAAA | 255 | 58 | NM_001113291.1 |
|  | R: GAGGTATCAGCCCACAGTAGA |  |  |  |

**Table S5.** Oligonucleotides sequence in this study.

| **Fragment name** | **Sequences (5’-3’)** |
| --- | --- |
| si-*ALDH1A1* | TACATGGGATCCTTAATTA |
